# Pharmacological Investigation of the Anti-Inflammation and Anti-Oxidation Activities of Diallyl Disulfide in a Rat Emphysema Model Induced by Cigarette Smoke Extract

**DOI:** 10.3390/nu10010079

**Published:** 2018-01-12

**Authors:** Yan Liu, Ang Li, Xiuli Feng, Xiao Sun, Xiaosong Zhu, Zhongxi Zhao

**Affiliations:** 1 School of Pharmaceutical Sciences, Shandong University, 44 West Wenhua Road, Jinan 250012, China; 18396865987@163.com (Y.L.); liangliang0725@aliyun.com (A.L.); fengxl0330@163.com (X.F.); sunxiaosdu@163.com (X.S.); 18766198651@163.com (X.Z.); 2 Shandong Engineering & Technology Research Center for Jujube Food and Drug, 44 West Wenhua Road, Jinan 250012, China; 3 Shandong Provincial Key Laboratory of Mucosal and Transdermal Drug Delivery Technologies, Shandong Academy of Pharmaceutical Sciences, 989 Xinluo Street, Jinan 250101, China

**Keywords:** diallyl disulfide, emphysema, redox imbalance, anti-inflammation, antioxidation

## Abstract

Diallyl disulfide (DADS) is the main organosulfur ingredient in garlic, with known antioxidant and anti-inflammatory activities. The aim of the present study was to investigate the effect of DADS on reducing the inflammation and redox imbalance in a rat emphysema model that was induced by intraperitoneal injection of cigarette smoke extract (CSE). Briefly, DADS exerted an anti-inflammation effect on emphysema rats through decreasing cell influx in the bronchoalveolar lavage fluid (BALF) and suppressing pro-inflammation cytokine production including tumor necrosis factor alpha (TNF-α), interleukin-1β (IL-1β), interleukin-6 (IL-6) via inhibiting the NF-κB pathway. In addition, levels of oxidative stress markers including malondialdehyde (MDA) and myeloperoxidase (MPO) were reduced, while the activities of glutathione (GSH), glutathione peroxidase (GSH-PX), superoxide dismutase (SOD) and total antioxidant capacity (T-AOC) were markedly enhanced by DADS. Moreover, MMP-9 and TIMP-1 expression were down-regulated by DADS. Furthermore, the regulation effects of DADS on CD4^+^ and CD8^+^ T cells were observed. In conclusion, these encouraging findings suggest that DADS could be considered as a promising anti-inflammation and antioxidative agent for the treatment of emphysema.

## 1. Introduction

Chronic obstructive pulmonary disease (COPD), as a major worldwide health problem, is characterized by persistent airflow limitation which is usually progressive and associated with an enhanced chronic inflammatory response in the airway and the lung to noxious particles or gases [1,2]. The World Health Organization (WHO) has estimated that it will increase to being the third leading cause of death, making this a global epidemic by 2020 [3].

The pathogenesis of COPD includes chronic inflammation, an imbalance of protease/anti-protease activities, oxidative stress, apoptosis, and autoimmune mechanisms [4,5,6,7]. Cigarette smoking is considered the major cause of COPD, which can induce oxidative stress, trigger pulmonary inflammation and immune dysregulation, followed by activating epithelial cells and macrophages, releasing inflammatory mediators such as tumor necrosis factor alpha (TNF-α), interleukin-1β (IL-1β), interleukin-6 (IL-6) and matrix metallo-peptidases (MMPs), and finally leading to inflammatory processes in airways, progressive airflow limitation and cell injury and apoptosis [8,9,10,11,12]. Thus, researchers have been focusing on intervening in the inflammatory processes of COPD by inhibiting different steps of the molecular and cellular pathways involved. It is plausible that agents with anti-oxidative, anti-inflammatory and immune regulation activities might be promising for the prevention and treatment of COPD.

To date, a glucocorticoid has been the first-line medicine for the treatment of COPD by systemic or inhaled medication. The newest inhalation therapies, such as inhaled corticosteroids and β_2_-agonists, provide patients with different choices. However, the inhalation of corticosteroids can increase the risk of pulmonary and oropharynx fungal infection, while the systemic application of corticosteroids can induce systemic immunity impairment, and increase the risk of more adverse events such as infection, steroid diabetes, and osteoporosis [13]. Therefore, researchers are committed to finding new treatments for COPD with better curative effects and less side effects.

Garlic possesses nutritional values and medicinal characteristics. Diallyl disulfide (DADS) is the main organosulfur ingredient in garlic, and has shown diverse pharmacological properties such as anti-inflammation [14,15], anti-oxidation [16,17,18,19,20] and immune regulation [21]. The anti-inflammatory effects of DADS observed can be attributed to its ability to decrease inflammatory mediators by inhibiting NF-κB translocation and i-κB phosphorylation, as well as suppressing the expression of mRNA for IL-1β and TNF-α [22,23]. In addition, the mechanism by which DADS protects against oxidative stress could be concluded as inducing the activation of the HO-1/Nrf-2 pathway, increasing Nrf2 nuclear translocation, as well as reducing reactive oxygen species (ROS), lipid peroxidation, Bax/Bcl-2 ratio, caspase-3 activation, and phosphorylation of JNK and P38 [16,17]. Diallyl disulfide (DADS) is also identified as the main highly volatile ingredient in a fork garlic respiratory therapy discovered in China that has shown encouraging clinical efficacy in the treatment and prevention of COPD and lung cancer [24].

In the present study, the pharmacological effects of DADS and the underlying mechanism on the treatment of COPD were determined. A rat emphysema model was established by intraperitoneal injection of cigarette smoke extract (CSE). The antioxidative and anti-inflammatory effects of DADS were explored by examining the antioxidant defense and oxidative stress biomarkers and inflammatory mediators. Besides this, a morphology assessment, Western blotting analysis for protein expressiones of NF-κB p65, i-κB, Nrf2 and NQO1, immunohistochemistry for expressiones of matrix metalloproteinase-9 (MMP-9), tissue inhibitors of metalloproteinases-1 (TIMP-1), CD4^+^ and CD8^+^ T cells in lung tissues were also performed.

## 2. Materials and Methods

### 2.1. Animals

Male SPF Sprague-Dawley rats (weight range, 120–140 g) were purchased from the Laboratory Animal Center of Shandong University (Grade II, Certificate No. SCXY 20090001, Shandong, China). Rats were housed in individual stainless steel cages and maintained in a controlled environment (temperature of 22–27 °C, a daily temperature variation ≤ 3, a relative humidity of 50–70%, and a 12/12 h light-dark cycle). All procedures were approved by the guidelines of the Ethical Committee Experimental Animal Center of Shandong University (No. 2016020, Jinan, China).

### 2.2. Reagents

DADS (Purity of 99%) was purchased from Tengzhou wutong aroma chemicals Co., Ltd. (Tengzhou, Shandong, China). Total superoxide dismutase (T-SOD), Malondialdehyde (MDA), glutathione peroxidase (GSH-PX), glutathione (GSH), myeloperoxidase (MPO) and total antioxidant capacity (T-AOC) commercial reagent kits were purchased from Nanjing Jiancheng Biology Engineering Institute (Nanjing, Jiangsu, China). Enzyme-linked immuno sorbent assay (ELISA) commercial kits for TNF-α, IL 1β and IL-6 were bought from Shanghai MultiSciences (Lianke) Biotech Co., Ltd. (Shanghai, China). DAB Detection Kit was purchased from Zhongshan Goldebridge Biotechnology CO., Ltd. (Beijing, China). Anti-NF-κB p65, i-κB, and NQO1 antibodies were purchased from Cell Signaling Technology (Danvers, MA, USA). Anti-Nrf2 antibodies were provided by Santa Cruz Biotechnology (Santa Cruz, CA, USA). Anti-GAPDH antibodies were provided by Proteintech Biotechnology (Rocky Hill, CT, USA). Horseradish peroxidase (HRP)-conjugated antibodies was bought from Jackson Immuno Research Laboratories, Inc. (West Grove, PA, USA). Rabbit polyclonal antibodies against rat CD4^+^, CD8^+^, MMP-9 and TIMP-1 were purchased from Abcam (Shanghai, China). Other reagents used in the experiment were all of analytical grade.

### 2.3. Preparation of Cigarette Smoke Extract (CSE)

Cigarette smoke extract (CSE) was prepared by using the previously reported method [25]. Briefly, three cigarettes (Taishan New, China Tobacco Shandong Industrial CO. Ltd., Jinan, China, tar, 11 mg; nicotine, 1.1 mg; carbon monoxide, 11 mg) were burned, and then the smoke was collected using a peristaltic pump, and finally bubbled into 10 mL phosphate-buffered saline (PBS). The CSE was freshly prepared before use and filtered through a 0.22-μm filter to remove particles and bacteria.

### 2.4. Animal Model

The rat emphysema model was established by intraperitoneal injection of CSE as previously described [25,26,27]. Rats were randomly selected and divided into five groups with six rats per group: blank control, DADS control, CSE, budesonide (positive drug) and DADS groups.

Each rat in the CSE group was intraperitoneally injected with 1 mL CSE-PBS solution on days 1, 8, and 15. Rats in the DADS and budesonide groups were intraperitoneally injected with 1 mL of CSE on days 1, 8, and 15 along with the daily injection of DADS or budesonide. The doss of test groups were as follows: 100 and 10 mg/kg/day for DADS and budesonide, respectively. DADS and budesonide injection solutions were freshly prepared by dissolving them in the injection vehicle of PBS containing 20% hydroxypropyl-β-cyclodextrin (*w*/*v*). Rats from the blank and DADS control groups without CSE received an intraperitoneal injection of the blank vehicle and DADS in the vehicle, respectively. The DADS control group used was to evaluate the influence of DADS on normal rats. All rats were fed under a same rearing condition for 21 days. On the 21st day after the experiment, all rats were sacrificed and harvested.

### 2.5. Preparation and Analysis of Bronchoalveolar Lavage Fluid (BALF)

Total and differential inflammatory cell counts in bronchoalveolar lavage fluid (BALF) were performed.

Rats were intraperitoneally injected with 10% chloral hydrate for euthanasia at the dose of 3 mL/kg. Bronchoalveolar lavage was performed on the right lung while the left lung was clamped for further preparation.

Immediately after euthanasia, the chest of each rat was opened to collect the BALF. The trachea and the right lung were cannulated and perfused with 2 mL ice cold normal saline. A three-in and three-out pattern of main bronchial instillation was adopted and the BALF was collected with a high recovery (70–80%). BALF samples were kept on ice to avoid cell lysis and the total volumes of BALF were recorded. The total cell number in the BALF was counted with an erythrocytometer. The BALF was then immediately centrifuged for 10 min at 1500 rpm and 4 °C. Pelleted cells were then resuspended with normal saline, and differentially counted by Wright–Giemsa staining. A total of 200 leukocytes were counted in each BALF sample, and the percentage of macrophage, neutrophil, and lymphocyte was calculated.

### 2.6. Sampling the Lung Tissue and Homogenization

The left middle lobe and lower lobe of lung were immediately frozen in liquid nitrogen until they were homogenized. The liver and spleen of each rat were collected and dried with filter paper and then weighted.

The tissue homogenates (10%, *w*/*v*) were prepared in normal saline as follows. The lung tissues (about 0.1 g) were subsequently homogenized in 1 mL normal saline and then centrifuged for 10 min at 3500 rpm and 4 °C. The supernatant was collected, and the content of protein was measured using the bicinchoninic acid method (BCA). The samples were stored in a freezer (−80 °C) for biochemical analyses.

### 2.7. Hematoxylin and Eosin Stain for the Morphology Assessment of Lung Tissues

For histological analyses, the left upper lobes were fixed in 10% (*v*/*v*) neutral buffered formalin and then embedded in paraffin. After being sectioned at 4 μm thickness, paraffin sections of lung tissue were stained with hematoxylin and eosin (HE) solution. The mean linear intercept (MLI) and destructive index (DI) of lung tissues were measured.

Mean linear intercept (MLI) is a measurement of the mean inter-alveolar septal wall distance to assess the airspace enlargement, and is widely used to indicate the average size of alveoli. MLI was assessed in 10 random and non-overlapping fields per lung by light microscopy at 100× magnification. Cross lines were drawn in a field excluding the vessels and bronchus and the total number of alveolar septa of the two lines was counted. The total length of the cross lines divided by the number of intercepts provides the MLI, that is, MLI = total length/number of alveolar septa, as described previously [28]. The destructive index (DI) is used to indicate the percentage of destroyed alveoli and estimate lung parenchymal destruction. The DI is quantified by dividing the number of destroyed alveoli by the total number of counted alveoli. Alveoli lying underneath the counting points were evaluated for the presence of destruction. A destructive alveolus was defined if at least one of the following alveoli conditions was observed: at least two alveolar wall defects, at least two intraluminal parenchymal rags in alveolar ducts, clearly abnormal morphology, or classic emphysematous changes [29].

### 2.8. Enzyme-Linked Immunosorbent Assay (ELISA) for Inflammatory Mediators

Tumor necrosis factor alpha (TNF-α), interleukin-1β (IL-1β) and interleukin-6 (IL-6) levels in lung homogenate were detected using commercial ELISA kits according to the protocols of the manufacturers.

### 2.9. Antioxidant Defense and Oxidative Stress Biomarkers in Lung Homogenate

The levels of Glutathione (GSH), glutathione peroxidase (GSH-PX), superoxide dismutase (SOD), total antioxidant capacity (T-AOC), malondialdehyde (MDA) and myeloperoxidase (MPO) in lung homogenate were measured using commercial kits (Nanjing Jiancheng Bioengineering Institute, Nanjing, China) according to the manufacturers’ instructions.

### 2.10. Western Blotting Analysis for Expression of Proteins

A total of 50 μg protein was separated on SDS-PAGE gel (Beyotime, Shanghai, China). Then, the proteins were transferred to the nitrocellulose (NC) membranes (Millipore, Burlington, MA, USA). Subsequently, the membranes were incubated with blocking solution (5% skim milk in TBST) for 1 h. After blocking, these membranes were washed three times with TBST and then incubated with specific antibodies against NF-κB p65, i-κB, Nrf2 and NQO1 (1:1000) as well as anti-GAPDH (1:3000) over night at 4 °C, respectively.

Next, these membranes were thoroughly washed three times with TBST and then incubated with horseradish peroxidase (HRP)-conjugated antibodies for 2 h at room temperature. After thoroughly washing with TBST, the membranes were then developed with enhanced chemiluminescence (ECL) detection (Amersham Bioscience, Bath, UK).

### 2.11. Immunohistochemistry for the Expression of CD4^+^, CD8^+^ T cells, MMP-9 and TIMP-1

The expression of CD4^+^, CD8^+^ T cells, MMP-9 and TIMP-1 in lung tissues of rats was determined by immunohistochemistry. The primary antibodies were rabbit polyclonal antibody, corresponding to rat CD4^+^, CD8^+^, MMP-9 and TIMP-1. All antibodies were applied at the 1:200 dilution. Immunohistochemistry was conducted according to the manufacturer’s instructions.

Briefly, the paraffin that was embedded with a lung tissue slice was deparaffinized and then rehydrated. Antigen was retrieved using sodium citrate and with heat-induced retrieval. After blocking with goat serum, anti-CD4^+^, CD8^+^, MMP-9 and TIMP-1 antibodies were applied overnight at 4 °C, respectively. Then horseradish peroxidase-conjugated second antibody was applied, and the expression of CD4^+^, CD8^+^, MMP-9 and TIMP-1 was finally visualized using a DAB detection kit. DAB staining suggested that cytoplasm of CD4^+^/CD8^+^/MMP-9/TIMP-1 positive expression was brown. Images of CD4^+^, CD8^+^, MMP-9 and TIMP-1 were obtained and photographed under microscope (Olympus Corporation, Tokyo, Japan).

### 2.12. Statistical Analysis

Graphpad Prism 5.0 (GraphPad Software, San Diego, CA, USA) was adopted to perform all statistical analyses. The values of quantitative and semi-quantitative analysis are quantified as mean ± standard error of the mean (SEM). Comparisons between two groups were conducted with an unpaired two-tailed Student’s *t*-test. Multiple group comparisons were performed with analysis of variance (ANOVA) followed by a *t*-test with the Bonferroni adjustment. A *p*-value less than 0.05 was considered statistically significant.

## 3. Results

### 3.1. General Observation

Before the experiment, differences in hair appearance, diet, activity, and reaction to the surrounding environment among rats were not obvious. However, the rats were in manic anxiety state immediately after intraperitoneal injection of CSE for about 1 h, followed by weakness and fatigue. Moreover, in the late stage of the experiment, rats in the CSE group were shriveled with yellow hair and slow reactions and movement. No animal died during the experiment.

### 3.2. Body Weight and Spleen/Liver Index

**Figure 1:**
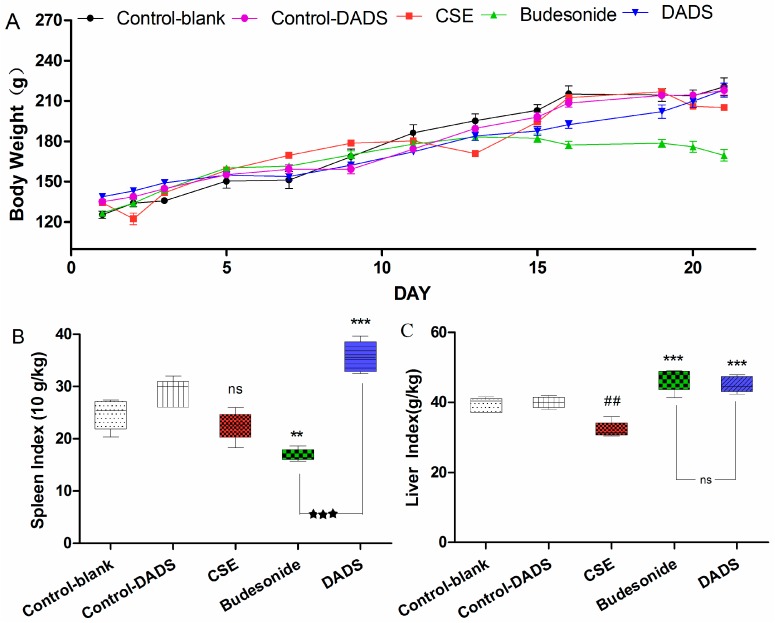
Effect of diallyl disulfide (DADS) on body weight (**A**); spleen index (**B**) and liver index (**C**) of rats in all groups. Rats (six animals per group) were treated by intraperitoneal injection as follows: cigarette smoke extract (CSE) group (1 mL CSE- phosphate buffer solution on days 1, 8, and 15), DADS group (100 mg/kg/day DADS + CSE), budesonide group (10 mg/kg/day budesonide + CSE), blank control group (the vehicle), and DADS control group (100 mg/kg/day DADS). (**A**) Rat body weights were measured in each group at the indicated time point; (**B**) The spleen index of rats in each group is indicated. Weight (spleen) g × 10/Weight (body) kg; (**C**) The liver index of rats in each group is indicated. Weight (spleen) g/Weight (body) kg. Data are presented as mean ± standard error of the mean (SEM) (*n* = 6/group). ## *p* < 0.01 vs. the blank control group. ** *p* < 0.01 and *** *p* < 0.001 vs. the CSE group. ^★★★^ *p* < 0.001 vs. the budesonide group. ^ns^ not significantly *p* > 0.05.

Rat survival quality can be partly reflected by body weight. The differences in body weight of rats among the groups were obvious after treatment for 21 days (Figure 1A). It is worth noting that the body weight of rats in the budesonide group was significantly reduced compared to the blank control group (169.48 ± 10.20 g of budesonide group vs. 220.42 ± 14.77 g of the blank control group, *p* < 0.001). In addition, it was found that the body weight of rats decreased in the CSE group relative to the blank control group (207.08 ± 3.65 g of the CSE group), but this was not statistically significant (*p* > 0.05). However, there was no significant difference of body weights in the DADS group (218.00 ± 11.51 g) compared to blank control group (*p* > 0.05). Besides, no statistically significant difference was found between the blank and DADS control groups (*p* > 0.05).

Spleen index is an immune parameter which is closely related to immune function and is usually used for immune function evaluation [30]. It was demonstrated that after treatment with budesonide, the spleen index was significantly reduced compared to the CSE group (Figure 1B). On the contrary, the spleen index was markedly increased after treatment with DADS compared to the CSE and budesonide groups. Moreover, the increase of spleen index in the DADS group was more obvious than that in the budesonide group (*p* < 0.001). The spleen index in the DADS control group was higher than that in the blank control group, but the difference was not statistically significant (*p* > 0.05).

As we can see in Figure 1C, a decrease in the liver index of the rats was observed in the CSE group compared with the blank control group, while an increase was found in both the budesonide and DADS groups. However, there was no significant difference in liver index values between the budesonide and DADS groups. In addition, there was no statistically significant difference between the blank and DADS control groups (*p* > 0.05).

### 3.3. Morphological Findings of Lung Tissues

**Figure 2:**
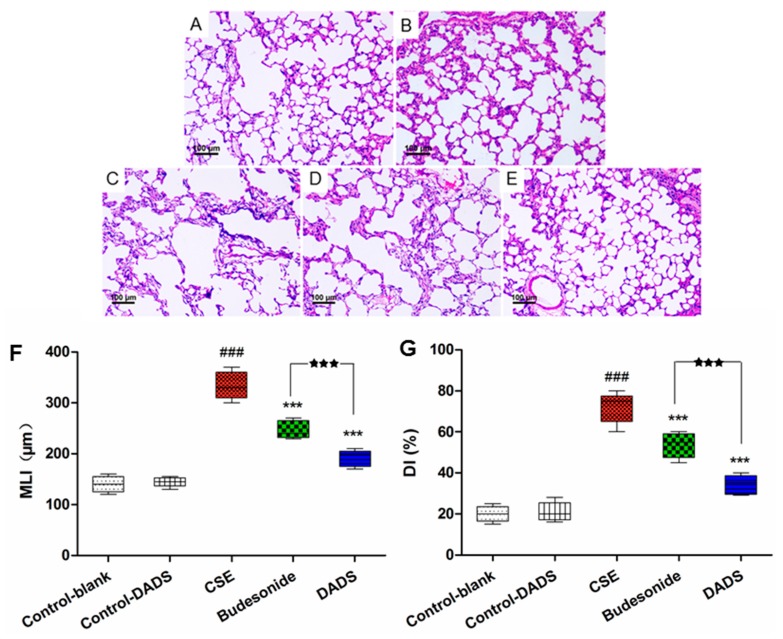
Effect of DADS on histological changes in lung tissue of CSE-induced emphysema in rats stained with hematoxylin and eosin (HE) (100× magnification). (**A**) Blank control group; (**B**) DADS control group (**C**) CSE group; (**D**) Budesonide group and (**E**) DADS group; (**F**) Morphometric measurements of the mean linear intercept (MLI) (μm) and (**G**) Destructive index (DI) (%). ### *p* < 0.001 vs. the blank control group; *** *p* < 0.001 vs. the CSE group; ^★★★^ *p* < 0.001 vs. the budesonide group.

The morphometric assessment was performed in order to evaluate lung damage in the treated rats. All HE-stained lung tissue slices were observed under a light microscope (100×). As expected, compared to the normal alveolar architecture (blank control group; Figure 2A), enlargement of the alveolar air spaces and destruction of the lung parenchyma were observed in the CSE group (Figure 2C), which indicated that the intraperitoneal injection of CSE in the rats caused lung destruction within 21 days. We observed an alleviation of emphysema in both the budesonide (Figure 2D) and DADS groups (Figure 2E) compared to the CSE group. Moreover, DADS treatment markedly reduced CS-induced pulmonary injury relative to the budesonide group.

The MLI and DI were significantly increased in the CSE group, while being decreased in the DADS and budesonide groups compared to the blank control group (*p* < 0.001). A significantly lower value of MLI and DI was observed in the DADS group than that in the budesonide group (*p* < 0.001) (Figure 2F,G). In addition, the difference of MDI and DI between the blank and DADS control groups was not statistically significant (*p* > 0.05).

### 3.4. Cellular Influx in BALF

**Figure 3:**
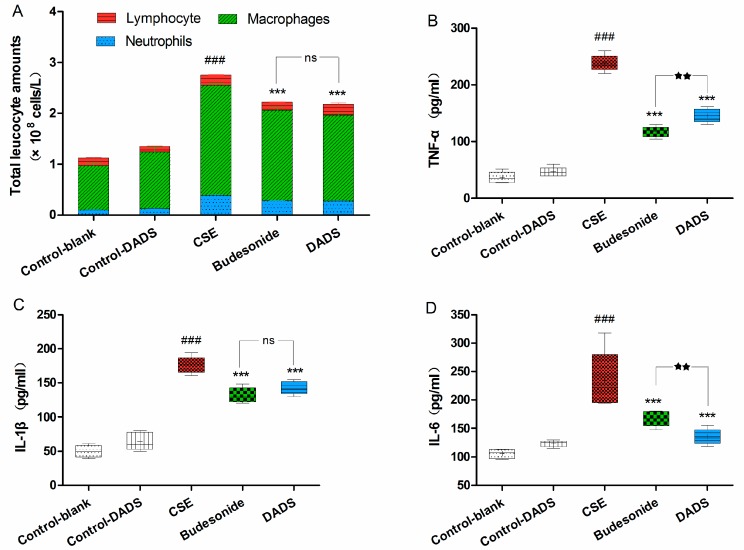
Evaluation of cell influx in the bronchoalveolar lavage fluid (BALF) and the levels of inflammation cytokines in lung homogenate. (**A**) Total cell number and the percentage of macrophage, neutrophil, and lymphocyte count in BALF. Levels of TNF-α (**B**); IL-1β (**C**) and IL-6 (**D**). Data are expressed as mean ± SEM, *n* = 6 rats per group. ### *p* < 0.001 vs. the blank control group; *** *p* < 0.001 vs. the CSE group; ^★★★^ *p* < 0.001 vs. the budesonide group; ^★★^ *p* < 0.01 vs. budesonide group; ^ns^ not significantly *p* > 0.05.

Macrophages, neutrophils, and lymphocytes are the main players in chronic immune inflammation in COPD. Exposure to CSE may induce prolonged airway inflammation relevant to the cellular infiltration of macrophages and neutrophils [31]. Therefore, we determined the number of total white blood cells as well as the differential count of neutrophils, macrophages and lymphocytes present in the BALF. There was no difference in BALF recovery volume among all groups. As shown in Figure 3A, a significant, nearly three-fold increase in the total white blood cell numbers in the BALF of the CSE group was observed compared to the blank control group (*p* < 0.001) As expected, the total number was markedly reduced in the BALF of rats from the budesonide and DADS groups (*p* < 0.001). Moreover, the differential count of neutrophils, macrophages, and lymphocytes in the BALF of the CSE group showed that CSE caused an obviously increased accumulation of neutrophils and macrophages, whereas there was a decrease in lymphocytes in the BALF. In addition, the macrophage and neutrophil counts of the DADS and budesonide groups were markedly reduced compared to the CSE group, although remaining obviously increased relative to the blank control group. However, there were no significant differences between the DADS and budesonide groups (*p* > 0.05). Furthermore, the total white blood cells, neutrophils, macrophages and lymphocytes between the blank and DADS control groups were not statistically significant (*p* > 0.05).

### 3.5. Evaluation of the Levels of Inflammatory Cytokines in Lung Homogenate

As we all know, smoking is related to inflammatory response. To investigate the possible effect of DADS on inflammation, we evaluated the levels of TNF-α, IL-1β and IL-6 in the lung homogenate (Figure 3B–D). As expected, we observed that there was an obvious increase in the levels of these three cytokines in the CSE group compared with the blank control group (*p* < 0.001). In contrast, treatment with DADS led to a marked decrease in the levels of these cytokines compared with the CSE group (*p* < 0.001). However, there was no statistically significant difference between blank and DADS control groups (*p* > 0.05). These results revealed that the administration of DADS alleviated the injury of airway inflammation and repressed pro-inflammatory cytokines in the lung tissue of the CSE-treated rats, while no effect on inflammatory cytokines was found in normal rats.

### 3.6. Effects of DADS on Oxidative Stress Markers (MPO, MDA) and Antioxidants (GSH, GSH-PX, SOD, T-AOC) in Lung Parenchyma

**Figure 4:**
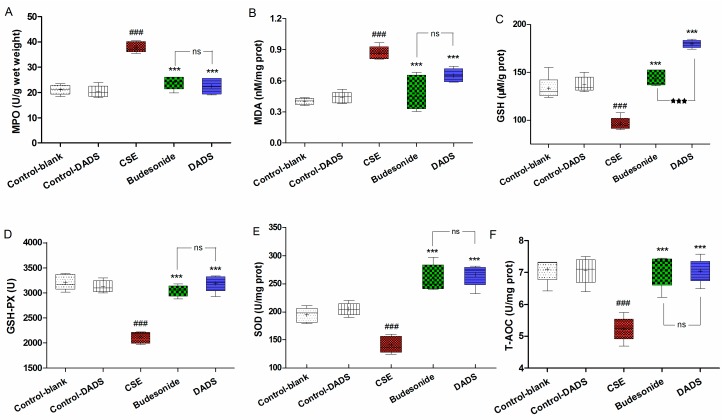
Effect of DADS on the oxidative stress markers and antioxidants in lung homogenate. Levels of myeloperoxidase (MPO) (**A**); Malondialdehyde (MDA) (**B**); Glutathione (GSH) (**C**); glutathione peroxidase (GSH-PX) (**D**); Superoxide dismutase (SOD) (**E**) and Total antioxidant capacity (T-AOC) (**F**). Data are expressed as mean ± SEM, *n* = 6 rats per group. ### *p* < 0.001 vs. the blank control group; *** *p* < 0.001 vs. the CSE group; ^★★★^ *p* < 0.001 vs. the budesonide group; ^ns^ not significantly *p* > 0.05.

The MPO, MDA, GSH, GSH-PX, SOD and T-AOC levels in the lung homogenate were determined to evaluate the effect of DADS on the oxidant/antioxidant imbalance that was induced by the influx of inflammatory cells. The MPO and MDA levels of the CSE group were markedly increased compared to the blank control group (*p* < 0.001) (Figure 4A,B). In contrast, rats from the budesonide and DADS groups had considerably lower MPO and MDA levels relative to the CSE group (*p* < 0.001). However, there was no significant difference in MPO levels among the budesonide and DADS groups (*p* > 0.05). As expected, the GSH, GSH-PX, SOD and T-AOC levels were considerably decreased in rats from the CSE group relative to the blank control group (*p* < 0.001) (Figure 4C–F). On the contrary, rats from the DADS and budesonide groups had higher levels of GSH, GSH-PX, SOD and T-AOC than the CSE group (*p* < 0.001). It is worth noting that the GSH activity was obviously induced after the DADS treatment (*p* < 0.001) (Figure 4C). Besides, the MPO, MDA, GSH, GSH-PX, SOD and T-AOC levels between the blank and DADS control groups were not statistically significant (*p* > 0.05), indicating that DADS had no effects on normal rats. Taken together with the data, it was evident that DADS down-regulated the oxidative stress markers and elevated the activities of antioxidants against the oxidant/antioxidant imbalance in the CSE-treated rats.

### 3.7. Effect of DADS on the Protein Expression of NF-κB p65, i-κB, Nrf2 and NQO1

**Figure 5:**
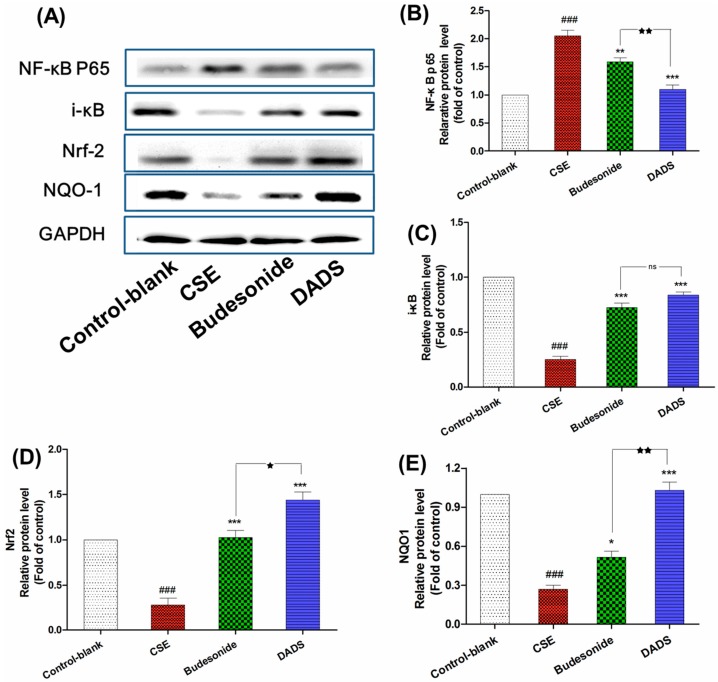
The effect of DADS on the protein expression of nuclear factor kappa B p65 (NF-κB p65), i-κB, nuclear factor erythroid 2-related factor 2 (Nrf-2), and NAD(P)H: quinone oxidoreductase 1 (NQO1) (**A**) Western blotting analyses of NF-κB p65, i-κB, Nrf-2, and NQO1 proteins; quantitative densitometric analyses of (**B**) NF-κB p65; (**C**) i-κB; (**D**) Nrf-2; and (**E**) NQO1 proteins normalized against glyceraldehyde-3-phosphate dehydrogenase (GAPDH). Each value represents the mean ± SEM of three independent experiments. ### *p* < 0.001 vs. the blank control group; * *p* < 0.05; ** *p* < 0.01 and *** *p* < 0.001 vs. the CSE group; ^★^ *p* < 0.05 and ^★★^ *p* < 0.01 vs. the budesonide group; ^ns^ not significantly *p* > 0.05.

NF-κB behaves as an important transcriptional repressor which can regulate many mediators of inflammation once actived by pro-inflammatory stimuli [32]. Nrf2 is a transcription factor which can protect against ROS triggered by smoking by binding to the antioxidant response element of important stress response genes [33]. Therefore, the protein expression of NF-κB p65, i-κB, Nrf2 and NQO1 was investigated via Western blotting analysis in order to evaluate the pathway by which DADS diverted its effect on CSE-treated rats (Figure 5A). As expected, the NF-κB p65 was obviously up-regulated in the CSE group in response to CSE induced inflammation while the expression of i-κB was markedly reduced in this group compared to the blank control group (*p* < 0.001) (Figure 5B,C). On the contrary, DADS reduced the level of NF-κB p65 as well as increasing that of i-κB compared to the CSE group (*p* < 0.001). As shown in Figure 5D,E, the level of Nrf2 and NQO1 showed a significant decrease after treatment in the CSE group compared to the blank control group (*p* < 0.001). However, DADS showed an obviously up-regulated effect on both of them compared to the CSE group. Moreover, DADS showed a much stronger effect on the expression of NF-κB p65, Nrf2 and NQO1 than budesonide (*p* < 0.05).

### 3.8. Immunohistochemistry for the Expression of the MMP-9, TIMP-1, CD4^+^ and CD8^+^ T cells

**Figure 6:**
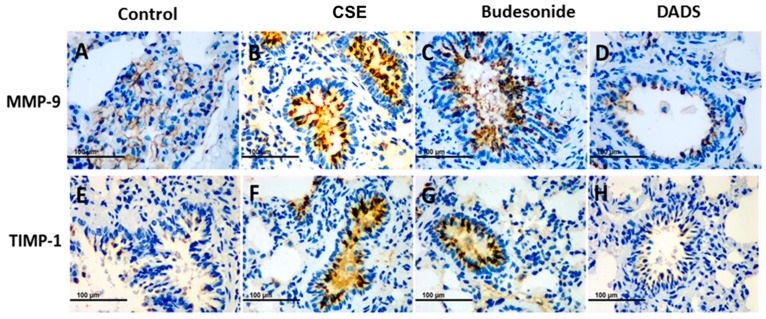
Immunohistochemistry analysis of the effect of DADS on the expression of matrix metalloproteinase-9 (MMP-9) and tissue inhibitor of metalloproteinases-1 (TIMP-1). Photomicrographs were taken at 400×. The expression of MMP-9 in (**A**) Blank control group; (**B**) CSE group; (**C**) Budesonide group and (**D**) DADS group as well as the expression of TIMP-1 in (**E**) Blank control group; (**F**) CSE group; (**G**) Budesonide group and (**H**) DADS group are shown.

It is known that MMP-9 and its inhibitor TIMP-1 play a central role in lung remodeling in COPD [34]. On the other hand, tobacco smoking stimulates the migration of CD4^+^ and CD8^+^ T cells into the damaged tissue and promotes the destruction of lung tissue [35,36]. The potential effect of DADS on MMP-9, TIMP-1, CD4^+^ and CD8^+^ T cells in lung tissue was investigated using immunohistochemical staining. All immunohistochemical staining lung tissue slices were observed under a light microscope (400×). Briefly, the accumulation of MMP-9 and TIMP-1 was significantly greater in the CSE group than the blank control group, while DADS showed an effective suppression of the expression. Furthermore, the inhibition effect of DADS was much stronger than that of budesonide (Figure 6).

**Figure 7:**
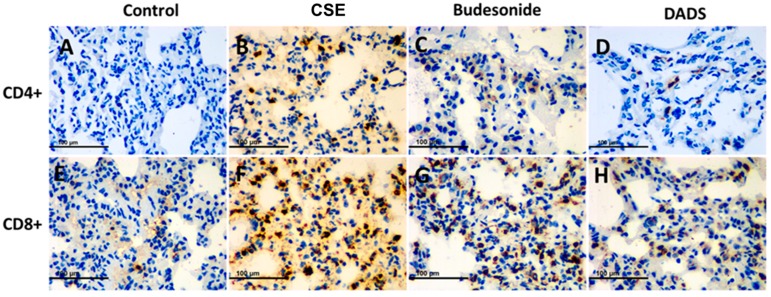
Immunohistochemistry analysis of the effect of DADS on the expression of CD4^+^ and CD8^+^ T cells. Photomicrographs were taken at 400×. The expression of CD4^+^ T cells in (**A**) Blank control group; (**B**) CSE group; (**C**) Budesonide group and (**D**) DADS group as well as the expression of CD8^+^ T cells in (**E**) Blank control group; (**F**) CSE group; (**G**) Budesonide group and (**H**) DADS group are shown.

As shown in Figure 7, DADS could effectively reduce the infiltration of CD8^+^ T cells in lung tissue compared to the CSE group. In all, our results indicate that DADS might participate in airway remodeling and immune regulation in the treatment of COPD.

## 4. Discussion

Inflammation, together with oxidative stress and protease-antiprotease imbalance in the airways, represents the typical pathology associated with chronic obstructive pulmonary disease (COPD) [4]. COPD-like airway injuries and a remodeling animal model can be established by chronic exposure to cigarette smoke [25]. In the present study, we established a rat emphysema model by cigarette smoking extract (CSE) injection in order to investigate the possible pharmacological effect of DADS on the CSE-treated rats. We observed several beneficial effects of DADS with regard to redox imbalance, inflammation, airway remodeling and immune regulation in the established rat emphysema model.

Rat survival quality can be partly reflected by body weight and spleen/liver index. In this study, more weight loss and smaller spleen index values in the budesonide group were observed compared to other groups, and these results were in line with a previous study that demonstrated that a significant reduction of body weight and spleen index occurred after systemic treatment with budesonide [37]. The spleen index of the DADS-treated group increased markedly in contrast to the blank control and CSE groups. Therefore, we hypothesized that the mechanism by which DADS exerted pharmacologic action might be partly due to immunostimulation.

Inflammation plays a central role in the development of COPD. The cellular infiltration of macrophages and neutrophils associated with prolonged airway inflammation can be induced by exposure to cigarette smoking (CS). Macrophages are derived from monocytes and are suggested to defend against noxious substances. A previous study showed that garlic can stimulate certain cell types such as macrophages, lymphocytes, natural killer cells and dendritic cells in order to enhance the functioning of the immune system [38]. In the present work, we observed that exposure to CS led to a high influx of macrophages and neutrophils into the BALF, whereas treatment with DADS decreased the amount of them. Moreover, a greater number of lymphocytes was found in the DADS treatment group than in the budesonide group, and this is in agreement with its previously reported immunomodulation effect [38]. Together with these results, we hypothesized that DADS might diversify its anti-inflammation effects by reducing the amount of macrophages as well as activating the adaptive immune response via stimulating lymphocytes.

Macrophages and neutrophils are also responsible for the production of pro-inflammatory cytokines including TNF-α, IL-1β and IL-6 [39]. TNF-α is an important pro-inflammatory factor that triggers a positive feedback loop during inflammation by activating NF-κB. Subsequently, the inflammatory process was amplified by the increase in TNF-α transcription [40]. IL-1β and IL-6 are important inflammatory mediators which are associated with lung inflammation, enlargement of distal airspaces, mucus metaplasia, and airway fibrosis [41,42]. After examining the cell influx in the BALF, we analyzed pro-inflammatory cytokines including TNF-α, IL-1β and IL-6 in lung tissue. Correspondingly, we found that that the pro-inflammatory cytokines were obviously decreased after DADS treatment.

Nuclear factor-κB (NF-κB) is a key transcription factor and regulates a number of genes involved in inflammation responses which can be enhanced by pro-inflammation stimuli such as TNF-α [43]. Our results demonstrated that DADS suppressed the transcriptional activity of NF-κB p65 and the degradation of its inhibitor i-κB.

The involvement of oxidative stress is related to chronic inflammation and periods of acute inflammation during exacerbation. Oxidative stress can be not only induced by cigarette smoking, but also results from the associated inflammatory response [33]. In order to clarify the antioxidant activity of DADS in rats, the levels of antioxidant enzymes (SOD and GSH-Px), as well as the contents of GSH, MDA, MPO and T-AOC, were investigated. Malondialdehyde (MDA) and MPO are both important oxidative stress markers. MDA, as a lipid peroxidation product, is an indicator of oxidative stress that is correlated inversely with pulmonary function [44]. MPO could increase the amount of ROS together with other oxidase and finally result in the irreversible lung damage [9,45]. Our results revealed that MDA and MPO levels were markedly reduced after treatment with DADS. Antioxidants can not only protect against the direct injurious effects of oxidants, but also alter the inflammatory events that play an important role in the pathogenesis of COPD [45]. Glutathione (GSH), as an efficient intracellular antioxidant of H_2_O_2_ which can be converted into GSSG by GSH-Px, plays an important role in the prevention of peroxidative lung damage in patients with COPD [46]. SOD functions as a scavenger of superoxide radicals in the body [45,46]. In this study, our data suggested that the activities of the major scavenger enzymes (SOD and GSH-Px) and the content of GSH were significantly decreased in rats from the CSE group, while the levels of MDA and MPO was obviously elevated compared to the blank control group. As expected, the DADS treatment markedly enhanced the levels of SOD, GSH-Px and GSH, while inhibiting the increase of MDA and MPO. In a word, these results indicated that DADS improved the enzymatic and non-enzymatic antioxidant defense systems against COPD.

Nrf2 plays an important role in protection against smoking-triggered ROS by the transcription of target genes involved in redox homeostasis, while NQO1 is a phase II detoxifying enzyme that can be regulated by Nrf2 [33]. In all, our Western blotting results revealed that Nrf2 protein expression was effectually enhanced, while protein expression of NQO1 was also up-regulated in the DADS group compared to the CSE group.

Matrix metalloproteases (MMPs) and tissue inhibitors of MMP (TIMP) imbalance plays a pivotal role in the destruction of lung parenchyma and the appearance of emphysema [47]. The enhanced release of MMP-9 by neutrophils and macrophages, which cannot be sufficiently counteracted by TIMP-1, is associated with the degradation of elastin in the alveolar walls and other extracellular matrix components and finally lead to lung destruction in COPD [48,49]. From the morphometric analysis, we observed a marked alleviation of emphysema in the DADS group. Therefore, we were prompted to investigate whether DADS has a regulation effect on the MMPS/TIMPs imbalance. In line with expectations, we found a significant increase of MMP-9 and TIMP-1 in rat lungs in the CSE group via immunohistochemical study. On contrary, the expression of MMP-9 and TIMP-1 in lung of rats was strongly suppressed by DADS compared to that in the CSE group.

The immune system plays an important role in COPD. Cigarette-smoking-induced chronic airway inflammation leads to the excessive recruitment of CD4^+^ and CD8^+^ cells as well as a diminished ratio of CD4^+^/CD8^+^ cells in the lung, which could be a consequence of the response to neoantigens [36,50]. CD8^+^ T cells exhibit cytotoxic activity toward antigens, while CD4^+^ T cells are critical for the induction and maintenance of CD8^+^ T cells [51]. Besides, CD4^+^ T cells exert immune activity by recruiting and activating innate immune cells such as natural killer cells and macrophages independently [52]. As reported, COPD is distinct from asthma with respect to the predominance of the CD8^+^ T-cell subset [53]. In the present study, we conducted an immunohistochemical study to evaluate the effect of DADS on the infiltration of CD4^+^ and CD8^+^ T cells in lung of rats. Our results suggested that DADS could effectively reduce the infiltration of CD4^+^ and CD8^+^ T cells in lung tissue compared to the CSE group. This is supported by a previous study which reported that the administration of aged garlic extract can decrease the number of CD4^+^ cells in the spleen [54].

Although glucocorticoids such as budesonide have played a key role in the treatment of COPD, patients are suffering severe side effects such as steroid diabetes, osteoporosis and glucocorticoid resistance [13]. In our study, the body weights and spleen indexes in the budesonide group were significantly decreased compared with the blank control group, whereas no changes were found in the DADS group. Furthermore, rats in the DADS control group did not show any statistically significant difference in body weight, liver/spleen index, inflammation cytokines and oxidative stress biomarkers. Therefore, it might be concluded that DADS could be a safer agent in the treatment of COPD than budesonide.

## 5. Conclusions

In conclusion, the potential pharmacological activity of DADS on rat emphysema, including anti-inflammation, anti-oxidation and immune regulation, was investigated in this study. Briefly, the results show a role for DADS as an anti-inflammation agent through decreasing cell influx and suppressing pro-inflammation cytokine production by inhibiting the NF-κB pathway. In addition, the mechanism by which DADS exerted anti-oxidation activities could be summarized as reducing oxidative stress markers as well as inducing antioxidative activities through activating the Nrf2 pathway. Moreover, MMP-9 and TIMP-1 expression was down-regulated by DADS. Finally, an immune regulation activity was also found in DADS by the regulation of the influx of CD4^+^ and CD8^+^ T cells. Based on these encouraging findings, DADS could become a promising agent for the treatment of COPD in the future.
